# Supplementary material for: Reporting trends and seasonality in the World Health Organization's Disease Outbreak News (DONs) across 1996–2023
Source: PLOS Glob Public Health. 2026 Apr 2;6(4):e0004876. doi: 10.1371/journal.pgph.0004876 (PMC13046118; doi:10.1371/journal.pgph.0004876)
Supplement: S1 Text — Table B. Observations of DONid “DON-1996-01-22-a”, with relevant variables selected to illustrate that a single report can contain information on outbreaks in different countries. Table C. Observations of DONid “DON-2010-10-25b”. In this Disease Outbreak News (DON) report, two disease outbreaks (Crimean-Congo haemorrhagic fever and dengue fever) were reported in Pakistan. Table D. An example of observations in the original DON dataset with duplicate links and different DONids. The incorrect entry was marked with a “Y” in the “Exclude” column and removed from the final dataset. Table E. Selected variables of interest which contain general information about the DON reports. Table F. Selected examples of DONs showing the difference between DiseaseLevel1 and DiseaseLevel2. Table G. The list of diseases featured in the DON dataset, by disease transmission group. Table H. Final negative binomial regression results for all DONs and DONs of the following transmission groups: Airborne and Droplet, Faecal-oral and FWB, and Vector-borne diseases. Table I. Final negative binomial regression results for all DONs and Airborne and Droplet transmission-related DONs, showing a comparison in model results for DONs before the hemisphere split and DONs of the Northern hemisphere. Table J. Final negative binomial regression results for all DONs, before and during the COVID-19 pandemic. Table K. Final negative binomial regression results for Airborne and Droplet transmission-related DONs, before and after the 2003 SARS-CoV-1 outbreak. (DOCX) [file pgph.0004876.s001.docx]

# Supporting Information

## List of R Packages Used

In addition to the packages loaded in R (version 4.2.2), the R packages used for the analysis are shown in Table A.

**Table A. List of R packages used.**

| **Name** | **Version** |
| --- | --- |
| conflicted | 1.2.0 |
| countrycode | 1.5.0 |
| dplyr | 1.0.9 |
| forcats | 0.5.1 |
| formula | 1.2.4 |
| ggmap | 4.0.0 |
| gridExtra | 2.3 |
| Hmisc | 4.7.1 |
| lattice | 0.20-45 |
| lmtest | 0.9-40 |
| lubridate | 1.8.0 |
| MASS | 7.3-60 |
| openxlsx | 4.2.5.2 |
| patchwork | 1.2.0 |
| purrr | 0.3.4 |
| readr | 2.1.3 |
| readxl | 1.4.1 |
| rnaturalearth | 0.1.0 |
| rnaturalearthdata | 0.1.0 |
| stringr | 1.4.0 |
| survival | 3.4-0 |
| TSA | 1.3.1 |
| tsibble | 1.1.3 |
| zeallot | 0.1.0 |
| zoo | 1.8-11 |

## Data Cleaning

### Removing Duplicates

In the original dataset, there are 3637 observations and 3088 DONids. Each DONid represents a DON report, following the format of “DON-YYYY-MM-DD”. Where more than one DON is reported on a date, Carlson et al. (2023) added a letter as a suffix to the DONid (e.g. “DON-1996-01-22-a”, “DON-1996-01-22-b”).

There may be multiple observations of the same DONid as a single DON may report outbreaks in different countries, or outbreaks of different pathogens. For example, Table B shows the entries in the DON database for “DON-1996-01-22-a”, a DON reporting cholera outbreaks in five different countries in 1996. In the dataset by Carlson et al. (2023), this report was entered as five observations. Each observation represents an outbreak in a different country (Cape Verde, Cote d’Ivoire, Iran, Iraq, and Senegal). The observations have the same DONid and DiseaseLevel1 (Cholera).

**Table B. Observations of DONid “DON-1996-01-22-a”, with relevant variables selected to illustrate that a single report can contain information on outbreaks in different countries.**

| **DONid** | **Headline** | **ReportDate** | **DiseaseLevel1** | **Country** |
| --- | --- | --- | --- | --- |
| DON-1996-01-22-a | Global Cholera Update | 1996-01-22 | Cholera | Cape Verde |
| DON-1996-01-22-a | Global Cholera Update | 1996-01-22 | Cholera | Cote d’Ivoire |
| DON-1996-01-22-a | Global Cholera Update | 1996-01-22 | Cholera | Iran |
| DON-1996-01-22-a | Global Cholera Update | 1996-01-22 | Cholera | Iraq |
| DON-1996-01-22-a | Global Cholera Update | 1996-01-22 | Cholera | Senegal |

A single DON report may also have information on different diseases. For example, Table C shows the entries for DONid “DON-2010-10-25-b”, a DON reporting two diseases (Crimean-Congo haemorrhagic fever and dengue fever) in Pakistan in 2010. In the DON dataset, these outbreaks are recorded as two separate observations.

**Table C. Observations of DONid “DON-2010-10-25b”. In this Disease Outbreak News (DON) report, two disease outbreaks (Crimean-Congo haemorrhagic fever and dengue fever) were reported in Pakistan.**

| **DONid** | **Headline** | **ReportDate** | **DiseaseLevel1** | **Country** |
| --- | --- | --- | --- | --- |
| DON-2010-10-25-b | Crimean-Congo haemorrhagic fever (CCHF) and Dengue in Pakistan | 2010-10-25 | Crimean-Congo haemorrhagic fever | Pakistan |
| DON-2010-10-25-b | Crimean-Congo haemorrhagic fever (CCHF) and Dengue in Pakistan | 2010-10-25 | Dengue fever | Pakistan |

Upon initial inspection of the dataset, it was observed that there were 3088 DONids but 3033 web links, suggesting that there were duplicate links (since each DONid should represent a unique link). To identify duplicates, the dataset was filtered for unique DONids with duplicate links. The list of DONs to be inspected were exported into an Excel sheet, and the links were manually checked for errors. Table D provides an example from the manual data check, where the DON report “1999 - Cholera in Kenya” was entered into the dataset twice. By viewing the original report, the observation of DONid “DON-1999-01-12” was identified as a duplicate of “DON-1999-01-20” and marked “Y” to be excluded. Table D shows an example of an entry, DONid “DON-1999-01-12”, which was marked for exclusion. Through manual verification by viewing the articles online, it was observed that the ReportDate of DONid “DON-1999-01-12” was incorrect, and the article was a duplicate of DONid “DON-1999-01-20”.

**Table D. An example of observations in the original DON dataset with duplicate links and different DONids. The incorrect entry was marked with a “Y” in the “Exclude” column and removed from the final dataset.**

| **DONid** | **Headline** | **ReportDate** | **Link** | **Exclude** | **ReasonForExclusion** |
| --- | --- | --- | --- | --- | --- |
| DON-1999-01-12 | 1999 - Cholera in Kenya | 1999-01-12 | https://www.who.int/emergencies/disease-outbreak-news/item/1999_01_20-en/ | Y | Duplicate of DON-1999-01-20. Incorrect date. |
| DON-1999-01-20 | 1999 - Cholera in Kenya | 1999-01-20 | https://www.who.int/emergencies/disease-outbreak-news/item/1999_01_20-en/ |  |  |

A total of 11 observations were excluded as they were duplicate entries of DON reports. For one of the entries, DONid “DON-2019-05-19”, the date was incorrectly scraped and was amended to “DON-2018-05-31”. After data cleaning, there were a total of 3077 unique DON reports in the DON dataset.

## Selection of Variables

Table E comprises definitions of the variables which were selected for the analysis.

**Table E. Selected variables of interest which contain general information about the DON reports.**

| **Disease transmission group** | **Definition** |
| --- | --- |
| DONid | A unique identification number for each report provided by Carlson et al. (2022) |
| Headline | The official headline of the DON report |
| ReportDate | The date of publication of the DON report on the official WHO website |
| Link | The web address on the official WHO website where the DON report can be accessed |
| DiseaseLevel1 | The name of the disease pertaining to the DON report |
| DiseaseLevel2 | A second variable for the disease name, where specific information on the disease is available (e.g., if “DiseaseLevel1” is “Influenza A”, “DiseaseLevel2” could contain information on the strain, “H5N1”) |
| Country | The country referenced in the DON report |
| ISO | The 3-letter ISO code of the country referenced in the DON report |

## Full Disease Classification

In the original DON dataset, two variables, “DiseaseLevel1” and “DiseaseLevel2”, were used by Carlson et al. (2023) to input data on diseases. “DiseaseLevel1'' contains information on the main disease names, while “DiseaseLevel2” includes information specific to the disease, where available. Examples of DONs which illustrate this difference are shown in Table F.

**Table F. Selected examples of DONs showing the difference between DiseaseLevel1 and DiseaseLevel2.**

| **DONid** | **Headline** | **ReportDate** | **DiseaseLevel1*** | **DiseaseLevel2†** |
| --- | --- | --- | --- | --- |
| DON-1996-02-15 | 1996 – Cholera in Africa | 1996-02-15 | Cholera |  |
| DON-1998-01-05 | 1998 – Influenza A(H5N1) in Hong Kong | 1998-01-15 | Influenza A | H5N1 |

* DiseaseLevel1 describes the main disease name of the outbreak described in the DON report.

† DiseaseLevel2 contains additional information about the disease, such as disease subtype. Not all entries in the DON dataset have a value in DiseaseLevel2.

Before classification, the list of diseases in “DiseaseLevel1'' were inspected to ensure that disease names were standardized. A new variable, “DiseaseLevelNew”, was created to contain the standardized disease names. Most of the disease names in “DiseaseLevel1” were replicated in “DiseaseLevelNew”, with exceptions: (i) “Syndromic: neurological” entries in “DiseaseLevel1” which indicated meningitis in “DiseaseLevel2” (“Meningitis: cerebrospinal”, “Meningitis: viral”, and “Meningitis”) were coded as “Meningitis” in “DiseaseLevelNew”; and (ii) entries which contained “Hemolytic uremic syndrome” in “DiseaseLevel1”, which were coded as “E. coli” in “DiseaseLevelNew”, since hemolytic uremic syndrome is a complication of E. coli infection. After standardizing disease names, a total of 78 distinct diseases were recorded in the “DiseaseLevelNew” variable. The full list of diseases in the DON categorized by disease transmission group are shown in Table G.

**Table G. The list of diseases featured in the DON dataset, by disease transmission group.**

| **Airborne and Droplet** | **Faecal-oral and FWB** | **Fluid-borne** | **STDs and Skin Contact** | **Vector-borne** | **Zoonoses** | **Other** |
| --- | --- | --- | --- | --- | --- | --- |
| Diptheria | Botulism | Crimean-Congo haemorrhagic fever | Gonorrhea | Chikungunya | Anthrax | Coccidioido-mycosis |
| COVID-19 | Cholera | Ebola virus | Hand, foot, and mouth disease | Dengue fever | Buffalopox | Elizabethkingia anophelis |
| Group A Streptococcus | Dysentery | Enterovirus | Human immunodeficiency virus | Dracunculiasis | Hantavirus | Guillain-Barre syndrome |
| Human coronavirus OC43 | E. coli | Lujo mammarenavirus | Monkeypox | Japanese encephalitis | Lassa fever | Syndromic: cardiovascular |
| Influenza A | Hemolytic uremic syndrome | Marburg fever | Staphylococcus | Leishmaniasis | Leptospirosis | Syndromic: diarrhoeal |
| Legionellosis | Hepatitis A | Transmissible spongiform encephalopathy |  | Malaria | Nipah virus | Syndromic: gastrointestinal |
| Measles | Hepatitis E |  |  | Mayaro virus | Plague | Syndromic: haemorrhagic |
| Meningococcal disease | Listeriosis |  |  | O'nyong-nyong fever | Rabies | Syndromic: hepatological |
| MERS-CoV | Polio |  |  | Oropouche fever | Rift Valley fever | Syndromic: neurological |
| Pertussis | Pseudomonas aeruginosa |  |  | St. Louis encephalitis | Streptococcus suis | Syndromic: respiratory |
| SARS-CoV-1 | Salmonella enterica |  |  | Tick-borne relapsing fever | Tularemia | Toxicity: bromide poisoning |
| Smallpox | Typhoid |  |  | West Nile virus | Typhus | Toxicity: lead poisoning |
| Tuberculosis |  |  |  | Western equine encephalitis | Venezuelan equine encephalitis | Toxicity: miscellaneous |
|  |  |  |  | Yellow fever |  | Unspecified |
|  |  |  |  | Zika virus disease |  |  |

Abbreviations: FWB, Food- and water-borne diseases; STDs, Sexually transmitted diseases.

## Final Negative Binomial Regression Results

The final negative binomial regression results for all DONs and DONs of Airborne and Droplet, Faecal-oral and FWB, and Vector-borne transmission groups, are shown in Table H.

**Table H. Final negative binomial regression results for all DONs and DONs of the following transmission groups: Airborne and Droplet, Faecal-oral and FWB, and Vector-borne diseases.**

| **Model** | **Regression type** | **Trend** | **Seasonality** | **Variable** | **Coefficient** | **Standard error** | **z value** | **p-value** | **95% CI** |
| --- | --- | --- | --- | --- | --- | --- | --- | --- | --- |
| All DONs | Negative Binomial | None | 12 mo | (Intercept) | 2.20 | 0.033 | 66.93 | <0.001 | (2.13, 2.27) |
|  |  |  |  | sin12 | 0.23 | 0.047 | 5.07 | <0.001 | (0.14, 0.33) |
|  |  |  |  | cos12 | -0.019 | 0.046 | -0.40 | 0.69 | (-0.11, 0.073) |
| Airborne and Droplet | Negative Binomial | None | 12 mo | (Intercept) | 1.40 | 0.058 | 24.11 | <0.001 | (1.29, 1.51) |
|  |  |  |  | sin12 | 0.59 | 0.082 | 7.14 | <0.001 | (0.42, 0.75) |
|  |  |  |  | cos12 | -0.13 | 0.081 | -1.64 | 0.1 | (-0.29, 0.025) |
| Faecal-oral and FWB | Negative Binomial | Negative | 9 mo | (Intercept) | 1.84 | 0.31 | 5.96 | <0.001 | (1.25, 2.42) |
|  |  |  |  | agg_date | -1.06e-04 | 2.15e-05 | -4.93 | <0.001 | (-1.47e-4, -6.57e-05) |
|  |  |  |  | sin9 | -0.37 | 0.089 | -4.19 | <0.001 | (-0.55, -0.20) |
|  |  |  |  | cos9 | -0.017 | 0.088 | -0.20 | 0.845 | (-0.19, 0.15) |
| Vector-borne | Negative Binomial | None | 12 mo | (Intercept) | 0.089 | 0.077 | 1.16 | 0.245 | (0.061, 0.24) |
|  |  |  |  | sin12 | 0.064 | 0.11 | 0.60 | 0.55 | (-0.14, 0.27) |
|  |  |  |  | cos12 | 0.50 | 0.11 | 4.60 | <0.001 | (0.28, 0.72) |

The final negative binomial regression results for all DONs and Airborne and Droplet transmission-related DONs, with a comparison in model results for DONs before the hemisphere split and DONs of the Northern hemisphere, are shown in Table I.

**Table I. Final negative binomial regression results for all DONs and Airborne and Droplet transmission-related DONs, showing a comparison in model results for DONs before the hemisphere split and DONs of the Northern hemisphere.**

| **Model** | **Regression type** | **Trend** | **Seasonality** | **Variable** | **Coefficient** | **Standard error** | **z value** | **p-value** | **95% CI** |
| --- | --- | --- | --- | --- | --- | --- | --- | --- | --- |
| All DONs | Negative Binomial | None | 12 mo | (Intercept) | 2.20 | 0.033 | 66.93 | <0.001 | (2.13,2.27) |
|  |  |  |  | sin12 | 0.23 | 0.047 | 5.07 | <0.001 | (0.14,0.33) |
|  |  |  |  | cos12 | -0.019 | 0.046 | -0.40 | 0.69 | (-0.11, 0.073) |
| All DONs  (Northern Hemisphere) | Negative Binomial | None | 12 mo | (Intercept) | 1.82 | 0.041 | 44.61 | <0.001 | (1.74, 1.90) |
|  |  |  |  | sin12 | 0.20 | 0.058 | 3.44 | <0.001 | (0.087, 0.31) |
|  |  |  |  | cos12 | 0.028 | 0.058 | 0.48 | 0.63 | (-0.087, 0.14) |
| Airborne and Droplet | Negative Binomial | None | 12 mo | (Intercept) | 1.40 | 0.058 | 24.11 | <0.001 | (1.29,1.51) |
|  |  |  |  | sin12 | 0.59 | 0.082 | 7.14 | <0.001 | (0.42,0.75) |
|  |  |  |  | cos12 | -0.13 | 0.081 | -1.64 | 0.10 | (-0.29,0.025) |
| Airborne and Droplet (Northern Hemisphere) | Negative Binomial | None | 12 mo | (Intercept) | 1.10 | 0.063 | 17.43 | <0.001 | (0.98,1.23) |
|  |  |  |  | sin12 | 0.61 | 0.089 | 6.83 | <0.001 | (0.43, 0.79) |
|  |  |  |  | cos12 | 0.31 | 0.089 | 3.47 | <0.001 | (0.14, 0.48) |

The final negative binomial regression results for all DONs, before and during the COVID-19 pandemic, are shown in Table J.

**Table J. Final negative binomial regression results for all DONs, before and during the COVID-19 pandemic.**

| **Model** | **Regression type** | **Trend** | **Seasonality** | **Variable** | **Coefficient** | **Standard error** | **z value** | **p-value** | **95% CI** |
| --- | --- | --- | --- | --- | --- | --- | --- | --- | --- |
| Before the COVID-19 Pandemic | Negative Binomial | None | 12 mo | (Intercept) | 2.25 | 0.035 | 65.00 | <0.001 | (2.19, 2.32) |
|  |  |  |  | sin12 | 0.22 | 0.049 | 4.49 | <0.001 | (0.13, 0.32) |
|  |  |  |  | cos12 | -0.031 | 0.049 | -0.63 | 0.527 | (-0.13, 0.066) |
|  |  |  |  |  |  |  |  |  |  |
| During the COVID-19 Pandemic | Negative Binomial | None | 12 mo | (Intercept) | 1.75 | 0.098 | 17.80 | <0.001 | (1.56, 1.94) |
|  |  |  |  | sin12 | 0.39 | 0.14 | 2.83 | 0.0047 | (0.12, 0.66) |
|  |  |  |  | cos12 | 0.17 | 0.14 | 1.21 | 0.23 | (-0.10, 0.43) |

The final negative binomial regression results for Airborne and Droplet transmission-related DONs, before and after the 2003 SARS-CoV-1 outbreak, are shown in Table.

**Table K. Final negative binomial regression results for Airborne and Droplet transmission-related DONs, before and after the 2003 SARS-CoV-1 outbreak.**

| **Model** | **Regression type** | **Trend** | **Seasonality** | **Variable** | **Coefficient** | **Standard error** | **z value** | **p-value** | **95% CI** |
| --- | --- | --- | --- | --- | --- | --- | --- | --- | --- |
| Before 2003 SARS-CoV-1 Outbreak | Negative Binomial | None | 12 mo | (Intercept) | 0.47 | 0.12 | 3.90 | <0.001 | (0.23, 0.71) |
|  |  |  |  | sin12 | 0.43 | 0.17 | 2.53 | 0.011 | (0.12, 0.76) |
|  |  |  |  | cos12 | -0.037 | 0.17 | -0.22 | 0.82 | (-0.39, 0.31) |
|  |  |  |  |  |  |  |  |  |  |
| After 2003 SARS-CoV-1 Outbreak | Negative Binomial | None | 12 mo | (Intercept) | 1.68 | 0.059 | 28.66 | <0.001 | (1.57, 1.80) |
|  |  |  |  | sin12 | -0.58 | 0.084 | -6.97 | <0.001 | (-0.75, -0.42) |
|  |  |  |  | cos12 | 0.087 | 0.083 | 1.05 | 0.29 | (-0.072, 0.25) |

## Calculation of Step-Change Difference

For the interrupted time series analyses, we obtained the step-change difference between models using the negative binomial regression model intercepts. For example, for the 2003 SARS-CoV-1 outbreak, a step-change difference of 3.77 reports was calculated. First, we extracted the intercept of the before and after models, at 0.47 and 1.68, respectively. At baseline where the periodicity terms are equal to zero, taking the exponent of the intercepts, the baseline of the number of reports before and after the 2003 SARS-CoV-1 outbreak was 1.60 reports and 5.37 reports, respectively. The equation for the negative binomial regression models is indicated below, with N representing the number of reports, $sin\frac{12\pi t}{T}$ and $cos\frac{12\pi t}{T}$ representing the periodicity terms where T is the fixed integer representing period, t is the integer representing the sequential month index, $\beta_{1}$ and $\beta_{2}$ representing the coefficients of the periodicity terms, and $\alpha$ representing the model intercept.

$$ln(N) =\beta_{1}sin\frac{12\pi t}{T}+\beta_{2}cos\frac{12\pi t}{T}+\alpha$$
